# Recreational Running Motivations among Breast Cancer Survivors

**DOI:** 10.3390/ijerph192315500

**Published:** 2022-11-23

**Authors:** Ewa Malchrowicz-Mośko

**Affiliations:** Department of Kinesiology, Faculty of Sport Sciences, Poznan University of Physical Education, 61-871 Poznan, Poland; malchrowicz@awf.poznan.pl

**Keywords:** runners, MOMS, cancer treatment, oncology patient, active lifestyle

## Abstract

Lifestyle-associated factors play an important role in prevention of such malignancies as breast cancer (BC), prostate cancer, or colon cancer. Physical activity (PA) before, during, and after diagnosis improves outcomes for BC. People after BC live with numerous side effects and PA has potential to reduce some of them. Unfortunately, few cancer survivors exercise regularly. The aim of this study was to ascertain motivations for running among BC survivors (in comparison with the motivations of healthy women) in order to better manage their attitudes in terms of PA and active lifestyle. A total of 317 Polish women took part in the study: 152 BC women (age 46.49 ± 7.83; BMI 24.78 ± 3.50) and 165 healthy runners (control group (age 36.91 ± 9.68; BMI 23.41 ± 3.94)) using the diagnostic survey method with the Motivation for Marathoners Scale (MOMS) questionnaire. Study results show that healthy runners had higher scores for health orientation, personal goal achievement, and affiliation compared to the group of BC survivors. The scores for weight concern, recognition, psychological coping, life meaning, and self-esteem were lower than those of BC survivors. These results should be included in the management of PA attitudes among BC survivors.

## 1. Introduction

The Motivation for Marathoners Scale (MOMS)—the diagnostic tool developed by Masters et al. [1]—has been widely used to ascertain motivations for running within different demographics, e.g., gender [2,3], age [4], nationality [5], or years of running experience [6]. Williams compared the health benefits of running versus walking in patients diagnosed with breast cancer (BC). Study results show that post-diagnosis running is associated with significantly greater reductions in BC mortality than post-diagnosis walking [7], but no up-to-date study has been conducted on why people with history of BC decide to run.

Lifestyle-associated factors play a significant role not only in cancer prevention: there is strong evidence that physical activity (PA) before, during, and after cancer diagnosis improves outcomes for BC. The findings suggest that PA protects against recurrence and progression in BC survivors [8]. In addition to hereditary and environmental factors, modifiable factors such as lifestyle, including insufficient level of PA and poor and improper diet, affect the incidence of BC and its recurrence. It is estimated that 9 in 10 cases of BC are due to non-genetic factors, and approximately 25% to 30% of total BC cases could be preventable with lifestyle interventions alone [9]. Obesity and weight gain are negative prognostic factors for BC survival. PA prevents weight gain and may decrease obesity [10]. There exists a relationship between prognosis and exercise, with risk reductions of 15–67% for BC-specific mortality and 18–67% for all-cause mortality [11]. Exercise is helpful for women during adjuvant therapy in early-stage BC [12]. Regular PA also reduces lymphedema in BC patients [13,14,15]. Many BC survivors lose muscle mass due to inactivity during intensive chemotherapy [16]. Resistance training and aerobic exercise improve cardiovascular and muscle-cell adaptation and increase peripheral blood flow. Hormone treatment used in BC patients may cause osteoporosis, but exercise promotes bone regeneration, decreasing osteoporosis levels. PA also reduces global low-grade inflammation, which is related with cardiovascular diseases that can be side effects of BC treatments [17]. It is crucial to maintain, promote, and improve the physical and mental conditions of BC patients. Unfortunately, studies suggest that many cancer survivors are insufficiently physically active. Older participants, women, and overweight or obese participants had significantly lower moderate-to-vigorous PA than their younger male normal-weight counterparts [8].

The number of BC survivors increases every year and women after BC live with numerous side effects. Exercise, as a safe and effective tool, reduces some of these side effects, but few cancer survivors are physically active [17,18,19]. Major determinants of low levels of exercise after BC are low pre-diagnosis levels of exercise, lower education, being postmenopausal, or having depressive symptoms [20]. Psychosocial barriers include low motivation and kinesiophobia, and perceived health benefits and social support/guidance by healthcare providers are significant facilitators. Inaccessible fitness facilities hinder cancer patient PA engagement, though the availability of tailored amenities appears to be a strong facilitator [21]. Interventions that facilitate interpersonal interaction and promote a sense of safety may be the most effective [22].

Patients treated with chemo-, radio-, immune-, and hormone therapy should be assisted in reaching recommended activity levels by targeted interventions during and after cancer therapy. In 2020, World Health Organization (WHO) issued official recommendations that adult cancer patients should perform the same weekly dose of PA as fully healthy people [23]. Almost 50% of cancer patients report cancer-related fatigue [17]. Regular exercise in BC survivors has been linked to reductions in cancer-related fatigue, nausea symptoms, and improvements in immune system function. PA has been recognized as an intervention to improve the quality of life in people with BC, build social relations after diagnosis, and combat stigmatization. Previous research has indicated relationships between PA and quality of life of patients with various malignant cancers. Physical exercises performed after mastectomy during treatment and after its completion also have a positive effect on the quality of their life. According to research, PA has a positive impact on the quality of life of cancer patients by affecting physical, functional, mental, emotional, and social well-being. The physical and functional benefits that have been demonstrated include improvements in functional capacity, muscular strength, flexibility, body composition, hematological indices, natural killer cell activity, nausea, fatigue, pain, and diarrhea. The psychological and emotional benefits include positive changes in personality functioning, locus on control, mood states including anxiety and depression, perceived physical competence, self-esteem, and satisfaction with life [24,25,26,27,28].

Exercise may be an effective intervention in reducing state anxiety in BC survivors, especially those with high state anxiety. Recreational activity decreases the risk of new primary cancer. Regular sport also protects patients against other comorbidities during and after oncology treatment. Exercise has many benefits for BC survivors, yet only half of this population regularly exercises (by WHO guidelines), and many oncology patients who exercised before their diagnosis do not return to the same exercise level [29,30,31,32,33,34,35].

The most frequent comorbidities among BC women are hypertension, diabetes, obesity, cardiovascular disease, and respiratory diseases [36]. The existence of these diseases increases with age, and 60–70% invasive BCs are diagnosed in women aged 55 years and older [37]. Some of the diseases can be treated with pharmacological drugs, but some, such as cancer-related fatigue, cannot [38]. Participation in rehabilitation is associated with an increase of PA after therapy [39]. It is acknowledged that low PA levels are associated with an increase in terms of both disease recurrence and mortality in cancer survivors. Running programs dedicated to oncological patients should consider intrinsic obstacles related to cancer and its treatment. The interventions should offer a personalized program performed by qualified trainers, together with a motivational approach able to improve participant adherence to an active lifestyle [40]. The aim of this study was to ascertain motivations for running among breast cancer survivors in order to better manage their attitudes in terms of PA and active lifestyle. Running has been recognized as a form of therapy that performs a number of important functions that are not only health-related, but also psycho-social (e.g., integration with other people, social recognition, relaxation) [41].

## 2. Materials and Methods

### 2.1. Study Participants

A total of 317 respondents, 152 BC women and 165 healthy women (control group) from Poland, participated in the online study using the diagnostic survey method with the Motivation for Marathoners Scale (MOMS) questionnaire. Only women 18+ took part in the online survey. All BC women were after hospital treatment (surgery, chemotherapy, or radiotherapy).

### 2.2. Research Tool

The diagnostic tool (MOMS) has been developed by Masters et al. in 1993 [1]. Answers to items on the MOMS questionnaire are on a seven-point Likert-type scale, where 1 meant Not a Reason, and 7 represented Most Important Reason. I used the Polish translation of the MOMS questionnaire, adapted and verified for reliability by Dybala [42]. The MOMS research tool contains 56 items. This scale shows nine dimensions or specific reasons for running, divided into a broader four groups of motives:

- Physical health: general health orientation (six items) and weight concern (four items).
- Achievement: personal goal achievement (six items) and competition (four items).
- Social motives: recognition (six items) and affiliation (six items).
- Psychological motives: psychological coping (nine items), self-esteem (eight items), and life meaning (seven items).

### 2.3. Statistical Analysis

**Figure 1:**
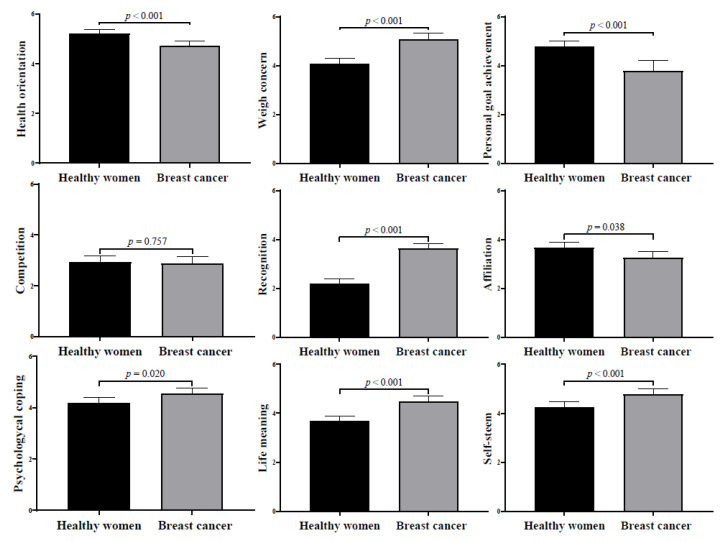
Differences for each of the MOMS dimensions in healthy runners and runners with breast cancer. Note. The reported β values are standardized coefficients. Covariates included were age and body mass index (kg/m^2^).

All analyses were performed with SPSS Statistics software package version 23.0. Initially, descriptive statistics (mean and standard deviation) for continuous variables were calculated. A Pearson correlation analysis was carried out to determine the relationship between all the study variables. Finally, a covariance analysis (ANCOVA) between healthy runners and BC survivors for each of the dimensions of the MOMS was conducted (Figure 1), after adjusting for age, and BMI.

## 3. Results

Table 1 shows the descriptive statistics of the study variables for healthy runners and BC survivors. Regarding socio-demographic characteristics, healthy runners were younger and had lower weight than BC survivors. The average weight of BC women (24.78) was close to the definition of BMI overweight (25.0). Regarding the MOMS dimensions, healthy runners had higher scores for health orientation, personal goal achievement, and affiliation compared to the group of BC survivors. Finally, the scores for weight concern, recognition, psychological coping, life meaning, and self-esteem were lower than those of BC survivors.

Table 2 presents the correlations between the study variables. The results showed a positive and significant relationship between all dimensions of the MOMS (all, *p* < 0.001), except for weight concern. A positive association was found between affiliation with recognition, psychological coping, life meaning, and self-esteem (all, *p* < 0.001). However, no relationship was found between weight concern with personal goal achievement, competition, and affiliation (all, *p* > 0.05).

Figure 1 displays the differences between each of the MOMS dimensions in healthy runners vs. BC survivors. Healthy runners showed higher motivation towards health (β = −0.49; 95% confidence interval (CI) = −0.76, −0.21; *p* < 0.001), personal goal achievement (β = −0.81; 95% CI = −1.12, −0.49; *p* < 0.001), and affiliation (β = −0.41; 95% CI = −0.80, −0.22; *p* < 0.05) compared to runners with BC. In contrast, runners with BC showed higher motivation in weight concern (β = 1.01; 95% CI = 0.65, 1.37; *p* < 0.001), recognition (β = 1.43; 95% CI = 1.14, 1.73; *p* < 0.001), psychological coping (β = 0.38; 95% CI = 0.06, 0.69; *p* = 0.020), life meaning (β = 0.80; 95% CI = 0.49, 1.11; *p* < 0.001), and self-esteem (β = 0.52; 95% CI = 0.22, 0.83; *p* < 0.001). No group difference was found for competition (β = −0.60; 95% CI = −0.43, 0.32; *p* = 0.757).

## 4. Discussion

The average weight of BC women (24.78) was close to the barrier of overweight (25.0). The average age of BC respondents was 46, the perimenopausal age. The most important motivations to run among BC survivors were these connected with weight (5.08). PA is very important in BC, especially in hormone-dependent tumors. PA is also especially important for BC women after menopause. Obesity plays an important role in the etiology of hormone-dependent (luminal) BCs in particular. While ovaries are the most important source of estrogen in premenopausal women, adipose tissue is the main producer in postmenopausal women, having a profound impact in ER-positive (estrogen-positive) BC incidence [43,44]. Weight gain affect almost 70% of cancer patients [17]. The weight problems are multifactorial [45] and overweight women exhibit a 30–40% increased risk of death. The problem of weight gain is also connected with increase of diabetes and cardiovascular disease risk [16,46].

Healthy women run mostly because of health orientation, personal goal achievement, and affiliation. This shows that personal goal achievement or desire to feel unity and integration with other people is not so important for cancer survivors. For BC women, weight concern, psychological coping, recognition, self-esteem, and life meaning are more important. Running and being active help them to have good opinions about themselves and to have better self-esteem. Running helps women with oncological disease in psychological coping and life meaning. In the difficult situation that living with a chronic and potentially fatal disease presents, being physically active is essential for improving the quality of life in the mental sphere for BC patients. At the time of cancer diagnosis and treatment, women may feel a sense of meaninglessness in life, and PA can help them survive hard times. History of stressful life events could be associated with a moderate increase in the risk of BC [47]. Managing stress is therefore a significant challenge for women with BC so as not to worsen their prognosis. Reduced stress and reduced risk of cancer recurrence are among the benefits of PA for BC survivors [48]. A cancer treatment is a good moment for supporting BC women in changing daily habits [49]. For the more individual approach, the objective is to know whether the BC patient has any cardiovascular diesase or other health problem, because it could impact the way they have to run [50]. Doctors, surgeons, and oncologists should inform BC patients about the positive effects of PA, such as running, for weight maintenance and stress management. Greater cooperation between oncologists, surgeons, sports psychologists, physiotherapists, cardiologists, diabetologists, sports instructors, personal trainers, and gym workers is required. The aspects of physical work with cancer patients should be widely taught at medical schools and universities of physical education.

It is necessary to add that the quantitative study of motivation for running using the MOMS scale focuses on identifying motivations and does not allow for a deep understanding of the reasons for participating in such a physically and mentally demanding task (after cancer). In the future, qualitative research should be conducted; for example, in-depth interviews with cancer survivors. This study only provides information about motivations to run in the middle of the lifespan: it is necessary to also check the motivations of younger runners and silver runners (seniors) after cancer. Moreover, health reasons were important motives for the investigated women; however, it is important to remember that according to research, the health literacy of recreational runners is rather low. Recreation runners know little about the health consequences of their PA and rely on colloquial claims [51]. Therefore, it is extremely important to also educate cancer patients about the possible health effects of running, and above all, about possible risks, especially for patients who suffer not only from cancer, but also from other comorbidities, e.g., diabetes, osteoporosis or cardiological diseases. In such cases, the level and quality of PA should be chosen in a very individual way. Finally, the issue of kinesiophobia should be checked during interviews with patients [52]. According to a previous study [51], younger runners have a slightly higher level of knowledge about the benefits and risks of running, so special attention should be paid to silver runners.

## 5. Conclusions

According to this study, women’s motivations for recreational running are different from the needs of healthy women. These differences should be taken into account when hospitals, physiotherapists, sports instructors or personal trainers create physical activity programs. Healthy women run mostly because of health orientation, personal goal achievement, and affiliation. For BC women, weight concern, psychological coping, recognition, self-esteem, and life meaning were more important. During the discharge of patients from the hospital, oncology centers should also organize lectures and conferences about cancer and lifestyle for patients.

## Figures and Tables

**Table 1:** Descriptive statistics of the sample of the study for healthy runners and BC survivors.

Measurements	Healthy Runners	Breast Cancer Survivors
	*M ± SD*	*M ± SD*
*n*	165	152
Sociodemographic characteristics		
Age (years)	36.91 ± 9.68	46.49 ± 7.83
Body mass index (kg/m^2^)	23.41 ± 3.94	24.78 ± 3.50
Health orientation (score: 1–7)	5.15 ± 1.37	4.80 ± 0.65
Weight concern (score: 1–7)	4.08 ± 1.87	5.08 ± 0.72
Personal goal achievement (score: 1–7)	4.97 ± 1.55	3.81 ± 0.93
Competition (score: 1–7)	3.18 ± 1.76	2.63 ± 1.32
Recognition (score: 1–7)	2.41 ± 1.35	3.43 ± 1.06
Affiliation (score: 1–7)	3.79 ± 1.92	3.10 ± 1.03
Psychological coping (score: 1–7)	4.27 ± 1.61	4.47 ± 0.74
Life meaning (score: 1–7)	3.77 ± 1.56	4.39 ± 0.71
Self-esteem (score: 1–7)	4.40 ± 1.55	4.64 ± 0.76

Note. *M*: medium; *SD*: standard deviation.

**Table 2:** Bivariate correlations among study variables.

Measurements	1	2	3	4	5	6	7	8	9	10	11
1. Age	-										
2. Body mass index	0.387 ***	-									
3. Health orientation	0.031	−0.018	-								
4. Weight concern	0.151 **	0.124 *	0.394 ***	-							
5. Personal goal achievement	−0.403 ***	−0.259 ***	0.356 ***	0.049	-						
6. Competition	−0.341 ***	−0.249 ***	0.195 ***	0.020	0.727 ***	-					
7. Recognition	−0.063	−0.088	0.209 ***	0.315 ***	0.248 ***	0.465 ***	-				
8. Affiliation	−0.248 ***	−0.158 **	0.207 ***	−0.097	0.444 ***	0.482 ***	0.368 ***	-			
9. Psychological coping	−0.071	−0.091	0.385 ***	0.280 ***	0.273 ***	0.252 ***	0.473 ***	0.360 ***	-		
10. Life meaning	−0.006	−0.028	0.419 ***	0.262 ***	0.271 ***	0.304 ***	0.590 ***	0.465 ***	0.808 ***	-	
11. Self-esteem	−0.137 *	−0.101	0.483 ***	0.366 ***	0.458 ***	0.428 ***	0.645 ***	0.411 ***	0.745 ***	0.783 ***	-

Note. * *p* < 0.05; ** *p* < 0.01; *** *p* < 0.001.

## Data Availability

The raw data supporting the conclusions of this article will be made available by the author, without undue reservation.

## References

[1] Masters K.S., Ogles B.M., Jolton J.A. (1993). The development of an instrument to measure motivation for marathon running: The Motivations of Marathoners Scales (MOMS). Res. Q. Exerc. Sport.

[2] Nikolaidis P.T., Chalabaev A., Rosemann T., Knechtle B. (2019). Motivation in the Athens Classic Marathon: The Role of Sex, Age, and Performance Level in Greek Recreational Marathon Runners. Int. J. Environ. Res. Public Health.

[3] Ogles B.M., Masters K.S., Richardson S.A. (1995). Obligatory running and gender: An analysis of participative motives and training habits. Int. J. Sport Psychol..

[4] Waśkiewicz Z., Nikolaidis P.T., Gerasimuk D., Borysiuk Z., Rosemann T., Knechtle B. (2019). What Motivates Successful Marathon Runners? The Role of Sex, Age, Education, and Training Experience in Polish Runners. Front. Psychol..

[5] Knechtle B., Rüst C.A., Rosemann T. (2013). The aspect of nationality in participation and performance in ultra-marathon running—A comparison between ‘Badwater’ and ‘Spartathlon’. OA Sport. Med..

[6] Malchrowicz-Mośko E., Gravelle F., Dąbrowska A., León-Guereño P. (2020). Do Years of Running Experience Influence the Motivations of Amateur Marathon Athletes?. Int. J. Environ. Res. Public Health.

[7] Williams P.T. (2014). Significantly greater reduction in breast cancer mortality from post-diagnosis running than walking. Epidemiology.

[8] Jochem C., Leitzmann M. (2022). Physical Activity and Sedentary Behavior in Relation to Cancer Survival: A Narrative Review. Cancers.

[9] Ortega M.A., Fraile-Martinez O., Garcia-Montero C., Pekarek L., Guijarro L.G., Castellanos A.J., Sanchez-Trujillo L., Garcia-Honduvilla N., Alvarez-Mon M., Bujan J. (2021). Physical Activity as an Imperative Support in Breast Cancer Management. Cancers.

[10] Irwin M.L., McTiernan A., Bernstein L., Gilliland F.D., Baumgartner R., Baumgartner K., Ballard-Barbash R. (2004). Physical activity levels among breast cancer survivors. Med. Sci. Sports Exerc..

[11] Betof A.S., Dewhirst M.W., Jones L.W. (2012). Effects and potential mechanisms of exercise training on cancer progression: A translational perspective. Brain Behav. Immun..

[12] Jones L.W., Peppercom J., Scott J.M., Battaglini C. (2010). Exercise therapy in the management of solid tumors. Curr. Treat Options Oncol..

[13] Schmitz K.H., Ahmed R.L., Troxel A., Cheville A., Smith R., Lewis-Grant L., Bryan C.J., Williams-Smith C.T., Greene Q.P. (2009). Weight lifting in women with breast-cancer-related lymphedema. N. Engl. J. Med..

[14] Torres Lacomba M., Yuste Sanchez M.J., Zapico Goni A., Prieto Merino D., Mayoral del Moral O., Cerezo Tellez E., Minayo Mogollón E. (2010). Effectiveness of early physiotherapy to prevent lymphedema after surgery for breast cancer: Randomized, single blinded, clinical trial. BMJ.

[15] Todd J., Scally A., Dodwell D., Horgaan K., Topping A. (2008). A randomized controlled trial of two programmes of shoulder exercise following axillary lymph node dissection for invasive breast cancer. Physiotherapy.

[16] Tredan O., Bajard A., Meunier A., Roux P., Fiorletta I., Gargi T., Bachelot T., Guastalla J.P., Lallemand Y., Faure C. (2010). Body weight change in women receiving chemotherapy for breast cancer: A French prospective study. Clin. Nutr..

[17] Casla S., Hojman P., Marquez-Rodas I., Lopez-Tarruella S., Jerez Y., Barakat R., Martin M. (2015). Running away from side effects: Physical exercise as a complementary intervention for breast cancer patients. Clin. Transl. Oncol..

[18] Galanti G., Stefani L., Gensini G. (2013). Exercise as a prescription therapy for breast and colon cancer survivors. Int. J. Gen. Med..

[19] Johansson A., Johansson A., Johansson K. (2013). Physical activity during and after adjuvant chemotherapy in patients with breast cancer. Physiotherapy.

[20] Schmidt M.E., Wiskemann J., Ulrich C.M., Schneeweiss A., Steindorf K. (2017). Self-reported physical activity behavior of breast cancer survivors during and after adjuvant therapy: 12 months follow-up of two randomized exercise intervention trials. Acta Oncol..

[21] Elshahat S., Treanor C., Donnelly M. (2021). Factors influencing physical activity participation among people living with or beyond cancer: A systematic scoping review. Int. J. Behav. Nutr. Phys. Act..

[22] Lynch A., Merdjanoff A., Wilson D., Chiarello L., Hay J., Mao J.J. (2022). Moving Forward: Older Adult Motivations for Group-Based Physical Activity After Cancer Treatment. Int. J. Behav. Med..

[23] (2020). World Health Organization Guidelines on Physical Activity and Sedentary Behavior.

[24] Kiebert G.M., Meyza J. (1997). Quality of life as a result of clinical trials in oncology—Selected issues. Quality of Life with Cancer.

[25] Courney K.S., Friedenreich C.M. (1999). Physical exercise and quality of life following cancer diagnosis: A literature review. Ann. Behav. Med..

[26] Courney K.S., McKey J.R., Jones L.W. (2003). Coping with cancer: Can exercise help?. Phys. Sportsmed..

[27] Czerniak U., Demuth A. (2010). Relationship between life quality perception and physical activity of females after mastectomy. Pol. J. Sport. Med..

[28] Czerniak U., Demuth A., Krzykała M., Ziółkowska-Łajp E. (2012). Body fat and quality of life in women treated for breast cancer. Stud. Phys. Cult. Tour..

[29] Sander A.P., Wilson J., Izzo N., Mountford S.A., Hayes K.W. (2012). Factors That Affect Decisions About Physical Activity and Exercise in Survivors of Breast Cancer: A Qualitative Study. Phys. Ther..

[30] Segal R., Zwaal C., Green E., Tomasone J.R., Loblaw A., Petrella T., Exercise for People with Cancer Guideline Development Group (2017). Exercise for people with cancer: A clinical practice guideline. Curr. Oncol..

[31] McNeely M.L., Campbell K.L., Rowe B.H., The Exercise for People with Cancer Guideline Development Group (2006). Effects of exercise on breast cancer patients and survivors: A systematic review and meta-analysis. CMAJ.

[32] Valenti M., Porzio G., Aielli F., Verna L., Cannita K., Manno R., Masedu F., Marchetti P., Ficorella C. (2008). Physical Exercise and Quality of life in breast cancer survivors. Int. J. Med. Sci..

[33] Yuan-Yuan L., Ho S.C., Cheung K.L., Yeo V.A., Lee R., Kwok C., Cheng A., Mo F., Yeo W. (2021). Higher Level of Sports Activities Participation during Five-Year Survival Is Associated with Better Quality of Life among Chinese Breast Cancer Survivors. Cancers.

[34] Blanchard C.M., Courneya K.S., Laing D. (2001). Effects of acute exercise on state anxiety in breast cancer survivors. Oncol. Nurs. Forum..

[35] Friedenreich C.M., Kopciuk G.J., Kopciuk K.A., Mackey J.R., Courneya K.S. (2009). Prospective cohort study of lifetime physical activity and breast cancer survivors. Int. J. Cancer.

[36] Kumar M., Nagpal R., Hemalatha R., Verma V., Kumar A., Singh S., Marotta F., Jain S., Yadav H. (2012). Targeted cancer therapies: The future of cancer treatment. Acta Biomed. Atenei Parm..

[37] American Cancer Society. www.cancer.org.

[38] Andrykowski M.A., Curran S.L., Lightner R. (1998). Off-treatment fatigue in breast cancer survivors: A controlled comparison. J. Behav. Med..

[39] Avancini A., Skrocke K., Tregnano D., Frada P., Trestini I., Cercato M.C., Bonaiuto C., Tarperi C., Schena F., Milella M. (2020). Running with cancer: A qualitative study to evaluate barriers and motivations in running for female oncological patients. PLoS ONE.

[40] Huy C., Schmidt M.E., Vrieling A., Chang-Claude J., Steindorf K. (2012). Physical activity in a German breast cancer patient cohort: One-year trends and characteristics associated with change in activity level. Eur. J. Cancer.

[41] Malchrowicz-Mośko E., Poczta J. (2018). Running as a form of therapy—Socio-psychological functions of mass running events for men and women. Int. J. Environ. Res. Public Health.

[42] Dybała M. (2013). Polska Adaptacja Kwestionariusza Motywów Biegaczy do Biegania/The Polish adaptation of the Motives of Runners for Running Questionnaire. Rozpr. Nauk..

[43] Friedenreich C.M., Shaw E., Neilson H.K., Brenner D.R. (2017). Epidemiology and Biology of Physical Activity and Cancer Recurrence. J. Mol. Med..

[44] Argolo D.F., Hudis C.A., Iyengar N.M. (2018). The Impact of Obesity on Breast Cancer. Curr. Oncol. Rep..

[45] Goodwin P.J., Ennis M., Pritchard K., McCready D., Koo J., Sidlofsky S., Trudeau M., Hood N., Redwood S. (1999). Adjuvant treatment and onset of menopause predict weight gain after breast cancer diagnosis. J. Clin. Oncol..

[46] Chlebowski R.T., Aiello E., McTiernan A. (2002). Weight loss in breast cancer patient management. J. Clin. Oncol..

[47] Bahri N., Fathi Najafi T., Homaei Shandiz F., Tohidinik H.R., Khajavi A. (2019). The relation between stressful life events and breast cancer: A systematic review and meta-analysis of cohort studies. Breast Cancer Res. Treat.

[48] Cuevas B.T., Hughes D.C., Parma D., Trevino-Whitaker R.A., Ghosh S., Li R., Ramirez A.G. (2014). Motivation, Exercise and Stress in Breast Cancer Survivors. Support Care Cancer.

[49] Blanchard C.M., Courneya K.S., Stein K. (2008). Cancer survivors’ adherence to lifestyle behavior recommendations and associations with health-related quality of life: Results from the American Cancer Society’s SCS-II. J. Clin. Oncol..

[50] Schmitz K.H., Courneya K.S., Matthews C., Denmark-Wahnefried W., Galvao D.A., Pinto B.M., Irwin M.L., Wolin K.Y., Segal R.J., Lucia A. (2010). American College of Sports Medicine roundtable on exercise guidelines for cancer survivors. Med. Sci. Sports Exerc..

[51] Stempień J.R., Stańczyk M., Tokarski J., Tkaczyk M. (2020). Running for health? Polish long-distance leisure runners and the problem of health literacy. Pol. Sociol. Rev..

[52] Malchrowicz-Mośko E. (2022). Kinesiophobia among Breast Cancer Survivors One Year after Hospital Treatment. Int. J. Environ. Res. Public Health.

